# A dual boundary classifier for predicting acute hypotensive episodes in critical care

**DOI:** 10.1371/journal.pone.0193259

**Published:** 2018-02-23

**Authors:** Sakyajit Bhattacharya, Vijay Huddar, Vaibhav Rajan, Chandan K. Reddy

**Affiliations:** 1 TCS Innovation Labs, Kolkata, India; 2 Amazon, Bangalore, India; 3 School of Computing, National University of Singapore, Singapore, Singapore; 4 Department of Computer Science, Virginia Tech, Arlington, United States of America; Clemson University, UNITED STATES

## Abstract

An Acute Hypotensive Episode (AHE) is the sudden onset of a sustained period of low blood pressure and is one among the most critical conditions in Intensive Care Units (ICU). Without timely medical care, it can lead to an irreversible organ damage and death. By identifying patients at risk for AHE early, adequate medical intervention can save lives and improve patient outcomes. In this paper, we design a novel dual–boundary classification based approach for identifying patients at risk for AHE. Our algorithm uses only simple summary statistics of past Blood Pressure measurements and can be used in an online environment facilitating real–time updates and prediction. We perform extensive experiments with more than 4,500 patient records and demonstrate that our method outperforms the previous best approaches of AHE prediction. Our method can identify AHE patients two hours in advance of the onset, giving sufficient time for appropriate clinical intervention with nearly 80% sensitivity and at 95% specificity, thus having very few false positives.

## 1 Introduction

**Fig 1:**
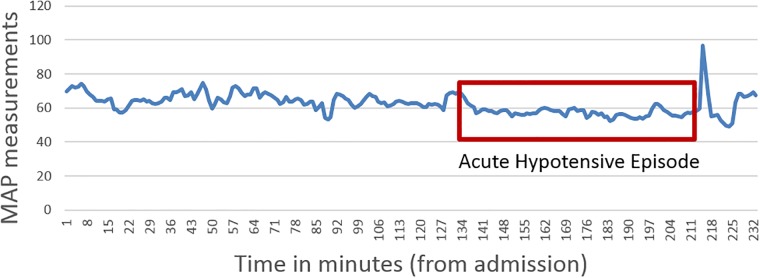
Definition of acute hypotensive episode: A period of 30 minutes where at least 90% of Mean Arterial Pressure measurements are no greater than 60 mmHg.

An Acute Hypotensive Episode (AHE) is the sudden onset of a period of sustained low blood pressure [1]. If left untreated, it can rapidly deteriorate a patient’s health and cause a number of complications including death. Fig 1 shows the episode in a graph of a patient’s Mean Arterial Blood Pressure (MAP) measurements.

Many different conditions may cause AHE; these include sepsis, myocardial infarction, cardiac arrhythmia, pulmonary embolism, hemorrhage, dehydration, anaphylaxis, effects of medication, or any of a wide variety of other causes of hypovolemia, insufficient cardiac output, or vasodilatory shock [1]. In many cases, AHE is a precursor to or predictor of other complications [2–4]. Determining the most appropriate medical intervention for such patients depends on first ascertaining the cause of the hypotensive episode: often due to lack of sufficient time, intervention begins by first selecting a suboptimal treatment to prevent immediate damage and to have sufficient time to assess the patient thoroughly [1]. Early identification of patients at risk for AHE helps not only in preventing the episodes but also in finding the best possible treatment for the patient [1].

Automated systems for predicting AHE and other complications in ICU patients are becoming increasingly important given the enormous difficulties of manually monitoring the abundance of data collected for each patient and the shortage of qualified clinical staff in ICUs [5, 6]. There have been many previous attempts to build automated systems that can identify patients at risk for AHE in advance (see section 2.2 for a brief survey).

This paper presents a novel supervised classification based system for identifying patients at risk for AHE. The algorithm uses only simple statistical summaries of past vital measurements (such as blood pressure and heart rate) of patients and can be employed in an online manner, facilitating real–time updates in the model and prediction. The novelty of the algorithm comes from learning to separate patient cases into three groups: (1) easy to recognize as AHE cases (2) easy to recognize as non–AHE cases and (3) ambiguous cases. During prediction, the algorithm deals with each of the cases in a different manner.

Our contributions in this paper are:

- A new supervised binary classification algorithm for identifying patients at risk for Acute Hypotensive Episodes. Our algorithm can be used both in offline and online modes. As more data from new patients arrive, the classifier updates itself in an online manner using only the newly available data (i.e. without re–training itself with the entire dataset).
- Extensive experiments on data from patients in critical care show that our algorithm achieves significantly higher accuracy, sensitivity and specificity compared to existing algorithms. Performance improvement is observed for predictions made up to 2 hours in advance, thus giving clinicians sufficient time for adequate medical intervention.

A preliminary version of this paper appeared in [7] where the dual boundary approach was first presented. A comparative analysis therein shows the superiority of our approach over state-of-the-art methods on a dataset of 1,700 patient records. In this paper, we provide a complete treatment of the most general online version of our algorithm (that was only briefly introduced earlier). We also analyze the behavior of the algorithm and reasons for its superior performance over baseline methods in detail. New experiments with additional baselines and data (vitals in addition to blood pressure) are presented. All experimental results in this paper are on a larger dataset of 4,593 patient records from the publicly available MIMIC II ICU database. To our knowledge, the only previously published work on dual boundary classifier is a preliminary version of this manuscript, as described above. Multiple decision boundaries may be obtained in margin-based classifiers, like SVM, and the difference between that and our algorithm is explained in section 4.1.

The rest of the paper is organized as follows. We begin with a brief background on AHE and a survey of related work in AHE prediction. Section 3 gives a formal definition of the problem. This is followed by a detailed discussion on our new classification algorithm in section 4. Details of the data and experimental protocol are presented in section 5 and section 5.5 describes the results obtained. We conclude our discussion in section 6.

## 2 Background

In this section we first provide a short description of blood pressure, hypotension and acute hypotensive episodes. This is followed by a brief survey of clinical studies on hypotensive episodes and related work in data mining on prediction of acute hypotensive episodes.

### 2.1 Acute hypotensive episodes

Arterial Blood Pressure (ABP) is the pressure exerted by blood on the vessels. Along with body temperature, respiratory rate and pulse rate, blood pressure is a vital sign that is routinely monitored by healthcare providers by measuring the pressure on the arterial walls.

Blood pressure values are measured often using a sphygmomanometer, in millimeters of mercury (mmHg). In each heartbeat blood pressure varies between the highest and the lowest pressure in the vessels, which are called systolic and diastolic pressures, respectively. ABP measurements are written as X/Y mmHg (e.g. 120/80) where X denotes the systolic pressure and Y denotes the diastolic pressure. The Mean Arterial Pressure (MAP) is the pressure generated as blood is pumped out of the left ventricle of the heart into the arteries. It can be approximated using the systolic (X) and diastolic (Y) pressures as follows [8]:

$$MAP=\frac{2}{3}X-\frac{1}{3}Y.$$

The normal range for blood pressure is considered to be between 90/60 and 130/80 mmHg [8]. Thus hypotension is the condition when the blood pressure is lower than 90/60 mmHg. Following [1], we define an Acute Hypotensive Episode as a period of 30 minutes during which at least 90% of the MAP measurements are no greater than 60 mmHg. Other guidelines have also been published [9, 10]. Commercial bedside monitoring systems provide alerts when the blood pressure falls below a predetermined level. But, to the best of our knowledge, there is no system that can predict AHE in advance.

### 2.2 Related work

Many clinical studies have examined the role of hypotension in complications like acute coronary syndromes [11], acute kidney injury [12] and sepsis [13, 14]. Hypotension is also found to be an important mortality predictor for patients with cardiovascular abnormalities [15, 16]. Hypotension is a precursor to septic shock, the second most common cause of death in ICU patients in the United States [17], and a study shows that mortality in such cases depends critically on the duration of hypotension before treatment [18].

Existing intervention approaches to AHE are reactive, that is, after the onset of AHE. These interventions include administration of vasopressors, fluid resuscitation and other treatments depending on the case [10, 19]. Instead if patients at risk for AHE are identified in advance, the intervention most appropriate to the patient can be determined and administered.

#### AHE prediction

In the MIMIC II critical care database [20], out of 2320 patients, AHE was found in 41% of the patients for whom arterial blood pressure was recorded. The mortality rate of these patients was found to be more than twice that of the entire MIMIC II population. Recognizing the importance of AHE, automated AHE prediction was put forth as a challenge in the 2009 Computers in Cardiology competition [1]. Vital signs data for 110 patients from the MIMIC II database [20] were provided, divided into training and test datasets containing 60 and 50 patient records respectively. The results of the top three performers of the challenge are published in [21], [22] and [23], respectively. The best result was obtained by Henriques et al [21] using a neural network based classifier. A similar method, called Chebyshev’s neural network, applied on the challenge dataset by Zhou et al [24], gave similar results. Simple mean based predictions such as those of Chen [22] who used the last 5 minutes of average diastolic blood pressure as a predictor of AHE and of Mneimneh et al [23] who used the last 20 minutes of MAP measurements had comparable performance. A hidden markov model based approach is proposed by Singh et al [25] and the use of contrast pattern mining for AHE prediction is discussed by Ghosh et al [26]. Both these techniques have performance similar to that of mean based predictions. Note that these methods were tested on a small sample size (110 patients) and for predicting AHE only 30 minutes in advance.

A detailed analysis of the problem as well as these methods is given in Marzyeh Ghassemi’s thesis [27] wherein the neural network based method is tested on 1,168 patient records using four vital signs’ data—heart rate, respiration rate, oxygenation levels and MAP. Sun et al [28] design a method based on particle swarm optimization and k-means to extract features from MAP measurements which are then classified using SVM. Donald et al [29] developed a Bayesian Artificial Neural Network trained on demographic and physiological data of nearly 2,000 patients to predict hypotension in brain injury subjects with observation windows of 15 and 30 minutes before the injury. Lee et al [30], used heart rate, MAP and clinical data of 1,311 patient records from the MIMIC II database. Their algorithm uses 102 statistical, wavelet-based and clinical features and predicts the event 1 hour in advance using a neural network based classifier.

Some other studies have also addressed the problem of predicting AHE but have not been tested extensively. A Binomial Sign Test based classifier was tested by Crespo et al [31] on the BP measurements of 7 patients using 5 time series features extracted from MAP waveforms. Their method predicts the event only 20 seconds in advance. Ghaffari et al [32] develop a method of detecting AHE using ECG and MAP waveforms and test it on 15 subjects from the MIMIC II database. Lehman et al [33] use a combination of Gaussian Mixture Model based clustering and K-Nearest Neighbours Classifier on 227 patient records from the MIMIC II database using both Heart Rate and MAP measurements. Rocha et al [34] use correlation analysis and neural networks on MAP measurements to build a predictive model for AHE. The best predictive accuracy when only vital measurements are used (among existing methods) is achieved by the mean-based prediction of Chen [22] which was shown to be outperformed by our classifier in [7].

## 3 Problem definition

An Acute Hypotensive Episode (AHE) is defined as a period of 30 minutes during which at least 90% of the MAP measurements are no greater than 60 mmHg [1]. We cast the problem of predicting AHE in a patient as a supervised binary classification problem. Using data from previous ICU patients—both with and without AHE events—a binary classifier is trained, where the classes are denoted by A (those likely to have AHE) and NA (those unlikely to have AHE). The classifier can then be used to predict AHE in a previously unseen patient by determining the class label of the patient. There are three parameters that can be varied during the prediction, which we will refer to as the [**P**,**O**,**T**] **parameters** of an algorithm:

**Prediction Window (P)**: Duration of time (in minutes) before which predictions are made, i.e., we predict whether or not a patient will have AHE *w* minutes in the future; we call *w* the *prediction window* width.
**Observation Window (O)**: Duration of time (in minutes) *before the prediction window* during which MAP measurements are considered for training the classifier.
**Test Window (T)**: Duration of time (in minutes) *before the prediction window* during which MAP measurements are considered for predicting AHE in a patient.

**Fig 2:**
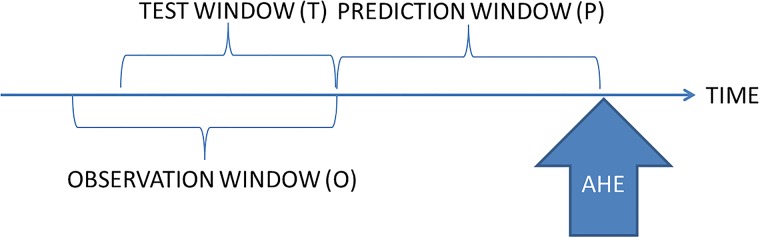
[O,P,T] parameters for a predictive algorithm. Note that the Test and Observation windows can vary in duration independently of each other.

Fig 2 shows a schematic of the three windows.

Note that each patient is potentially at risk of a future acute hypotensive episode at each point in time. What we have, from historical data, is a single indicator of an occurrence of AHE at a particular timepoint (the risk at previous timepoints can only be estimated, not known with certainty). Our aim is to predict this as early as possible.

The observation window indicates the amount of data we use for training. The test window indicates the amount of data we use during prediction. The windows can be measured with respect to either time duration (that we use) or number of measurements. For conducting our experiments, we vary the length of these windows (i.e. the time duration of the measurements considered for training and prediction) independently and study the performance of the classifiers. Also note that by testing the performance on expanding test windows, we effectively test the performance at regular intervals for the same patient. We also study the classifier performance in an online setting, where we update the classifier with new measurements obtained.

In a real-life deployment, the classifier can predict, with each new blood pressure measurement, whether a patient is at risk (A) or not at risk (NA) and when the prediction is A, an alert is raised for the clinical staff. So the labels can also be viewed as an indicator for alert at the patient level.

## 4 DBC: A novel classification method

In this section, we describe our new algorithm for classifying patients into classes 1 (e.g. class A, those at risk for developing AHE) and 2 (e.g. class NA, those not at risk for developing AHE) based on their blood pressure signals. Our method uses only the mean (*μ*_1_, *μ*_2_ for classes 1 and 2 respectively) and standard deviation (*σ*_1_, *σ*_2_, for classes 1 and 2 respectively) of the vital measurements within a given observation window as features. Our classifier, called Dual Boundary Classifier (**DBC**) uses a combination of margin-based and distance-based classification techniques. We first describe the intuition behind the algorithm and explain how it differs from standard classifiers like SVM and KNN.

### 4.1 Intuition behind the classifier

We observe in the training data that the MAP measurements in class A in the hour before the Acute Hypotensive event is generally lower than the MAP measurements in class NA. Hence, a margin-based classifier is expected to work well. As seen in the experiments, SVM has high specificity but its sensitivity is very low.

**Fig 3:**
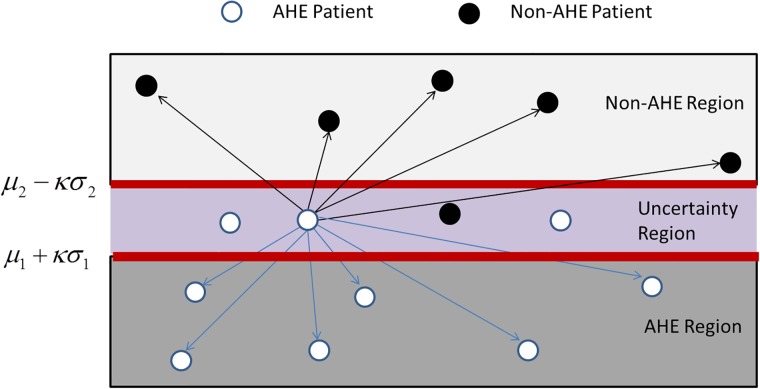
Schematic of our novel classifier. The feature space is divided into three regions based on the values of *μ*_1_, *μ*_2_, *σ*_1_, *σ*_2_, *κ*_0_. A test sample in the AHE region is classified into class A, Non-AHE region is classified into class NA. A distance-based approach is used for test samples in the Uncertainty region. The arrows for a point in the uncertainty region (shown only for one of the points), show that distances are calculated with respect points outside the uncertainty region only.

To minimize both false positives and false negatives (type-I and type-II errors, respectively), where true positive denotes correct identification in class A, our new method builds an interval-based margin (in other words, two decision boundaries, using a parameter *κ*). Our interval is based on the mean and standard deviation values in classes A and NA, observed in the training data: (*μ*_1_ + *κσ*_1_, *μ*_2_ − *κσ*_2_). Any value (in the test set) falling below the lower bound of the interval is considered to be in class A and a value above the upper bound in class NA. We choose the bounds (by choosing the right *κ*) such that the errors are minimized, i.e., the sum of the number of class A values falling above the lower bound (*n*_1_ + *n*_2_) and the number of class NA values falling below the upper bound (*n*_3_ + *n*_4_) is minimized. In addition we also minimize the number of values in the uncertainty region. Fig 3 shows a schematic.

Test samples falling in the region of class A or class NA are classified in a straightforward manner. For those test samples that lie in the uncertainty region, we use a distance-based approach to classify the sample by comparing the distance of the test case to (training data) points outside the uncertainty region.

#### Comparison with other classifiers

Note that our approach is not an ensemble-based technique since we do not use a combination of classifiers on any test datapoint. Our classifier is a discriminative classifier which applies two different classification rules and both rules differ in methodology from standard classifiers. One is a boundary based rule (similar to, but not the same as, SVM) for data points outside the uncertainty region, and another is a nearest neighbor rule (similar to, but not the same as, KNN) for datapoints in the uncertainty region. Thus the selection of the rule is also determined by our classifier in a data-driven manner. These differences distinguish DBC from standard classifiers.

*Comparison with margin-based classifiers*. The two boundaries in DBC are not determined using support vectors (as in SVM) but using the mean and standard deviation values of training data from both classes. In the optimization setup for training, DBC minimizes the misclassification rate and the number of points in the uncertainty region which is not the same as maximizing the margin. Indeed, our formulation yields a mixed integer linear program (shown in [7]) whereas the classical SVM formulation results in a quadratic program.

*Comparison with distance-based classifiers*. DBC uses a distance-based approach only for the uncertainty region points in testing and note that we determine the distance only with respect to the points outside the AHE region—which makes it different from a KNN rule. For example, if a test data point lies in the uncertainty region and the closest point is also within the uncertainty region, KNN will consider it whereas DBC will not.

To further illustrate the difference, note that a combination of SVM and KNN would apply both classifiers on each test point. But DBC first determines whether the test point lies in the uncertainty region. If it is in the uncertainty region, it applies a distance-based rule—checking distance only with respect to training points outside the uncertainty region. If it is not in the uncertainty region, then the boundary determines the class and the boundaries are learnt from the training data in a manner that is different from that of margin-based classifiers.

### 4.2 Online classification algorithm

We describe the DBC algorithm in the more general online mode. The offline mode is a special case with a single round that uses all available historical data for training. An implementation, in R, is available from the authors upon request.

The online algorithm proceeds in rounds. In each round, we use the measurements made in the new observation window to update the model. We denote the round with superscript (*n*). Measurements can be made for each of the vitals (subscript *v*): MAP, heart rate (HR), respiration rate (RR), Oxygen Saturation (OSAT) and temperature (TEMP).

#### Training the classifier

Training the classifier consists of the following steps:

- Denote by ${\text{y}}_{Av}^{\left(n\right)}$ the vector where each coordinate has the mean of the measurements of vital *v* in the *n*^*th*^ observation window for patients in class A. Let ${\text{y}}_{NAv}^{\left(n\right)}$ be the corresponding vector for class NA. Denote by ${\text{y}}_{Av}^{\left(n\right)}$ (${\text{y}}_{NAv}^{\left(n\right)}$) all the measurements of vital *v* for patients in class A (NA) until round *n*.
- Let $\mu_{Av}^{\left(n\right)}=\text{mean}\left({\text{y}}_{Av}^{\left(n\right)}\right)$, $\mu_{NAv}^{\left(n\right)}=\text{mean}\left({\text{y}}_{NAv}^{\left(n\right)}\right)$, $\sigma_{Av}^{\left(n\right)}=\text{stdev}\left({\text{y}}_{Av}^{\left(n\right)}\right)$, $\sigma_{NAv}^{\left(n\right)}=\text{stdev}\left({\text{y}}_{Av}^{\left(n\right)}\right)$, $\nu_{Av}^{\left(n\right)}=\text{variance}\left({\text{y}}_{Av}^{\left(n\right)}\right)$, $\nu_{NAv}^{\left(n\right)}=\text{variance}\left({\text{y}}_{Av}^{\left(n\right)}\right)$, are the corresponding means, standard deviations and variance.
- Compute the cumulative means and variance (for all measurements of vital *v* up to the *n*^*th*^ observation window), $\mu_{Av}^{\left(n\right)},\nu_{Av}^{\left(n\right)}$ as follows [35], where *x* is each measurement in ${\text{y}}_{Av}^{\left(n\right)}$:
  $$\begin{array}{c} \mu_{Av}^{\left(n\right)}=\mu_{Av}^{\left(n-1\right)}+\frac{x-\mu_{Av}^{\left(n-1\right)}}{n}\\ \nu_{Av}^{\left(n\right)}=\frac{\left(n-1\right)\nu_{Av}^{\left(n-1\right)}+\left(x-\mu_{Av}^{\left(n-1\right)}\right)\left(x-\mu_{Av}^{\left(n\right)}\right))}{n} \end{array}$$
  Similarly, we compute cumulative mean and variance $\mu_{NAv}^{\left(n\right)},\nu_{NAv}^{\left(n\right)}$ for class NA.
- Let $n_{Av}=\left|{\text{y}}_{Av}^{\left(n\right)}\right|$ and $n_{NAv}=\left|{\text{y}}_{Av}^{\left(n\right)}\right|$, the cardinalities of the two vectors. This is updated in each round:
  $$\begin{array}{ccc} n_{Av} & = & \left|{\text{y}}_{Av}^{\left(n-1\right)}\left|+\right|{\text{y}}_{Av}^{\left(n\right)}\right|\\ n_{NAv} & = & \left|{\text{y}}_{NAv}^{\left(n-1\right)}\left|+\right|{\text{y}}_{NAv}^{\left(n\right)}\right|. \end{array}$$
- Let *κ*_*v*_ be a value such that $\mu_{Av}^{\left(n\right)}+\kappa_{v}\sigma_{Av}^{\left(n\right)}<\mu_{NAv}^{\left(n\right)}-\kappa_{v}\sigma_{NAv}^{\left(n\right)}$. Let
  *n*_*v*1_: Number of ${\text{y}}_{Av}^{\left(n\right)}$ values greater than $\mu_{NAv}^{\left(n\right)}-\kappa_{v}\sigma_{NAv}^{\left(n\right)}$.
  *n*_*v*2_: Number of ${\text{y}}_{Av}^{\left(n\right)}$ values in the range $\left(\mu_{Av}^{\left(n\right)}+\kappa_{v}\sigma_{Av}^{\left(n\right)},\mu_{NAv}^{\left(n\right)}-\kappa_{v}\sigma_{NAv}^{\left(n\right)}\right)$.
  *n*_*v*3_: Number of ${\text{y}}_{NAv}^{\left(n\right)}$ values lesser than $\mu_{Av}^{\left(n\right)}+\kappa_{v}\sigma_{Av}^{\left(n\right)}$.
  *n*_*v*4_: Number of ${\text{y}}_{NAv}^{\left(n\right)}$ values in the range $\left(\mu_{Av}^{\left(n\right)}+\kappa_{v}\sigma_{Av}^{\left(n\right)},\mu_{NAv}^{\left(n\right)}-\kappa_{v}\sigma_{NAv}^{\left(n\right)}\right)$.
- Find the value of *κ*_*v*_ that minimizes *n*_*v*1_ + *n*_*v*2_ + *n*_*v*3_ + *n*_*v*4_. Let that value be $\kappa_{v}^{*}$. A simple heuristic for selecting $\kappa_{v}^{*}$ can be employed in practice:
  - Select a range of values for *κ*_*v*_ and compute *n*_*v*1_ + *n*_*v*2_ + *n*_*v*3_ + *n*_*v*4_ for each value in the range.
  - Select the value of *κ*_*v*_ with the (local) minimum *n*_*v*1_ + *n*_*v*2_ + *n*_*v*3_ + *n*_*v*4_.
  Alternatively, a mixed integer linear program can be used as shown in [7] but that can become computationally expensive especially in an online setting.
  Thus a trained classifier at the end of the *n*^*th*^ round consists of a 7-tuple: $\left[n_{Av},n_{NAv},\mu_{Av}^{\left(n\right)},\sigma_{Av}^{\left(n\right)},\mu_{NAv}^{\left(n\right)},\sigma_{NAv}^{\left(n\right)},\kappa_{v}^{*}\right]$ for each vital *v*.

#### Classifying test data

We compute the mean of vital measurements *v* over the period of the Test Window, denoted by *z*_*v*_. For each vital, we obtain a classification label *l*_*v*_ as follows:

- If $z_{v}<\mu_{Av}^{\left(n\right)}+\kappa_{v}^{*}\sigma_{Av}^{\left(n\right)}$, assign the patient to class A.
- If $z_{v}>\mu_{NAv}^{\left(n\right)}-\kappa_{v}^{*}\sigma_{NAv}^{\left(n\right)}$, assign the patient to class NA.
- If *z*_*v*_ falls in the range $\left[\mu_{Av}^{\left(n\right)}+\kappa_{v}^{*}\sigma_{Av}^{\left(n\right)},\mu_{NAv}^{\left(n\right)}-\kappa_{v}^{*}\sigma_{NAv}^{\left(n\right)}\right]$, compute the mean squared deviation of *z*_*v*_ from all the points in ${\text{y}}_{Av}^{\left(n\right)}$ and ${\text{y}}_{NAv}^{\left(n\right)}$ (from the training data), denoted by *d*_*Av*_ and *d*_*NAv*_, respectively. If *d*_*Av*_ > *d*_*NAv*_ assign the patient to class NA, otherwise assign the patient to class A.
  Note that this does not require us to store all the patient data used during the training phrase. The computation can be simplified as follows. The mean squared deviation of *z*_*v*_ from all the points in ${\text{y}}_{Av}^{\left(n\right)}$ is $\sum_{i=1}^{n_{Av}}{\left(z_{v}-x_{iv}\right)}^{2}$ where *x*_*iv*_ is the *i*-th coordinate of ${\text{y}}_{Av}^{\left(n\right)}$. The above expression can be written as
  $$d_{Av}=\sum_{i=1}^{n_{Av}}{\left(z_{v}-\mu_{Av}^{\left(n\right)}+\mu_{Av}^{\left(n\right)}-x_{iv}\right)}^{2}=n_{Av}[{\left(z_{v}-\mu_{Av}^{\left(n\right)}\right)}^{2}+\nu_{NAv}^{\left(n\right)}]$$
  and similarly for the distance of *z*_*v*_ from ${\text{y}}_{NAv}^{\left(n\right)}$. Thus the computation only requires the values of the total number of datapoints: *n*_*Av*_, *n*_*NAv*_ and the mean and variance computed during training: $\mu_{Av}^{\left(n\right)},\nu_{Av}^{\left(n\right)},\mu_{NAv}^{\left(n\right)},\nu_{NAv}^{\left(n\right)}$.

The final classification label is set by a majority voting rule: ∑_*v*_ *l*_*v*_ where ties are broken by using the vote for MAP.

### 4.3 Time complexity

The time complexity of training (for each round, and each vital) is dominated by the step of finding *κ*_*v*_. Let *K* be the number of choices for *κ*_*v*_ considered in our heuristic. The training time complexity is $O(K+n_{Av}+n_{NAv})$ and testing each test window *z*_*v*_ containing *n*_*z*_ vital measurements takes $O(n_{z})$ time (to compute the mean).

## 5 Experimental results

### 5.1 ICU data

MIMIC II [20] is a publicly available database, part of Physionet [36], containing physiological signals and clinical data of more than 5000 ICU patients. Vital signs of most of these ICU patients were recorded, sampled either every minute or every second. We consider only the following vital measurements for our study: Mean Arterial Blood Pressure (MAP), Heart Rate (HR), Pulse Rate (PUL), Respiratory Rate (RR) and Oxygen Saturation (OSAT).

Each patient record consists of a time series signal *x*_*t*_, *t* = 1,…,*N*_*i*_ where *N*_*i*_ is the total number of vital measurements for the *i*^*th*^ patient, and *x*_*t*_ is the MAP measurement at time *t*, time being reckoned from the start of the measurements in the ICU at an interval of 1 minute. Whenever the sampling is per second, we use the mean of all measurements in a minute. An Acute Hypotensive Event (AHE) is defined as an interval in the record, [*x*_*t*_, *x*_*t*+30_], in which at least 27 of the MAP measurements are no greater than 60.

Not all the records are usable since many of them have missing or erroneous data. From the available cleaned data in MIMIC II, we use 4,593 records of ICU patients out of which 1,307 are found to contain AHE events (class A) and 3,286 do not contain AHE events (class NA). We considered data only until the first AHE event in each patient record for training and prediction. During training we discard records that contain less than 6 hours of MAP measurements and those that contain Acute Hypotensive Events in the first 5 hours of recorded data.

### 5.2 Experimental setup

We test the performance of the algorithms for the following choices of 6 observation windows (O), 24 prediction windows (P) and 6 test windows (T):

$$\begin{array}{c} \text{O}\in \{20,40,60,80,100,120\},\\ \text{P}\in \{5,10,15,\ldots ,110,115,120\},\\ \text{T}\in \{20,40,60,80,100,120\}. \end{array}$$

In the online mode, for each of the 864 [O,P,T] settings, first approximately 20% (= 910) of the patient records are chosen for initial training. The ratio of AHE and Non-AHE patients in this initial training set is 260:650 which approximately maintains the same ratio found in the original data (1307:3286). After that we incrementally predict and update the classifier, using a single observation window for each patient. In this manner, prediction is done for the remaining 3,683 (= 4593-910) patients containing 1,047 AHE and 2,636 non-AHE cases. In the offline mode, we perform 5-fold cross validation over all the patient records, for each of the 864 [O,P,T] settings.

### 5.3 Baseline comparison methods

As mentioned earlier, among the previous methods for AHE prediction that use only vital measurements, the best predictive accuracy is achieved by the mean-based prediction of Chen [22] which we also confirm with experiments on our larger dataset of 1,700 patient records in our previous work [7]. We use this classifier, denoted by MU, as a baseline along with the other standard classifiers—Support Vector Machines (SVM) with RBF kernel, Random Forest (RF), K–Nearest Neighbors (KNN) and Adaboost (ADA)—using the same mean–based features. Implementations in R are used for these classifiers: packages ‘e1071’, ‘Random Forest’, ‘Class’ and ‘Ada’ respectively. Tunable parameters for the baseline classifiers are obtained in the following way. A portion of the training data is held out for validation. A grid search on the parameter space is performed and for each classifier, the parameters that give the best performance on the validation set are chosen. Prediction results are shown only for the best parameters thus selected.

### 5.4 Evaluation metrics

We measure the performance of all the tested methods by their sensitivity, specificity and classification accuracy. Sensitivity is defined as the percentage of class A test samples accurately classified into class A. Specificity is defined as the percentage of class NA test samples accurately classified into class NA. Classification accuracy of a classifier is the percentage of total test samples correctly classified.

Note that there is no discriminating threshold in our method that creates a trade-off between sensitivity and specificity (unlike many other classifiers). Hence the sensitivity and specificity do not change with either any internal parameter or time window. We show the sensitivity (true positive rate) and specificity (true negative rate) separately, where ‘positive’ denotes correct prediction of AHE.

P-values are computed using a one-sided two-sample t-test. We compare the mean value of the performance measure (sensitivity, specificity and accuracy) for a baseline (*μ*_0_) against the corresponding mean value for DBC (*μ*_1_). We compare the null hypothesis *H*_0_ : *μ*_1_ = *μ*_0_ against the alternative hypothesis *H*_*a*_ : *μ*_1_ > *μ*_0_ at level of significance 5%.

### 5.5 Performance results

The prediction window is the most important parameter here since it determines how early we can predict the possibility of AHE in a patient. The values of O and T parameters can be set by the user based on the sensitivity-specificity requirements or availability of data.

We first present the results of online and offline classification using only MAP measurements first. The results, shown in Table 1 are averages over all the 864 [O,P,T] window settings: averaged for 3683 patients in the online mode and over 5-fold cross validation in the offline mode as described above. We observe that our algorithm significantly outperforms all other algorithms (p-value <0.05) in terms of sensitivity, specificity and overall accuracy, in both online and offline modes.

**Table 1:** Mean (standard deviation in parentheses) of performance of algorithms—Our (DBC), KNN, RF, SVM, ADA and MU—Averaged over all the 864 [O,P,T] window settings: Averaged for 3,683 patients in the online mode and over 5-fold cross validation in the offline mode.

	Algorithm	DBC	KNN	RF	SVM	ADA	MU
Online	Sensitivity	**0.77 (0.04)**	0.31 (0.05)	0.30 (0.11)	0.17 (0.05)	0.24 (0.05)	0.39 (0.06)
	Specificity	**0.92 (0.02)**	0.47 (0.03)	0.37 (0.03)	0.82 (0.04)	0.60 (0.21)	0.81 (0.05)
	Accuracy	**0.85 (0.01)**	0.40 (0.03)	0.34 (0.06)	0.78 (0.07)	0.41 (0.07)	0.72 (0.07)
Offline	Sensitivity	**0.83 (0.01)**	0.34 (0.02)	0.43 (0.03)	0.19 (0.03)	0.39 (0.11)	0.59 (0.03)
	Specificity	**0.90 (0.01)**	0.56 (0.04)	0.34 (0.03)	0.78 (0.03)	0.57 (0.09)	0.79 (0.02)
	Accuracy	**0.87 (0.01)**	0.46 (0.03)	0.37 (0.03)	0.74 (0.04)	0.44 (0.08)	0.69 (0.02)

**Fig 4:**
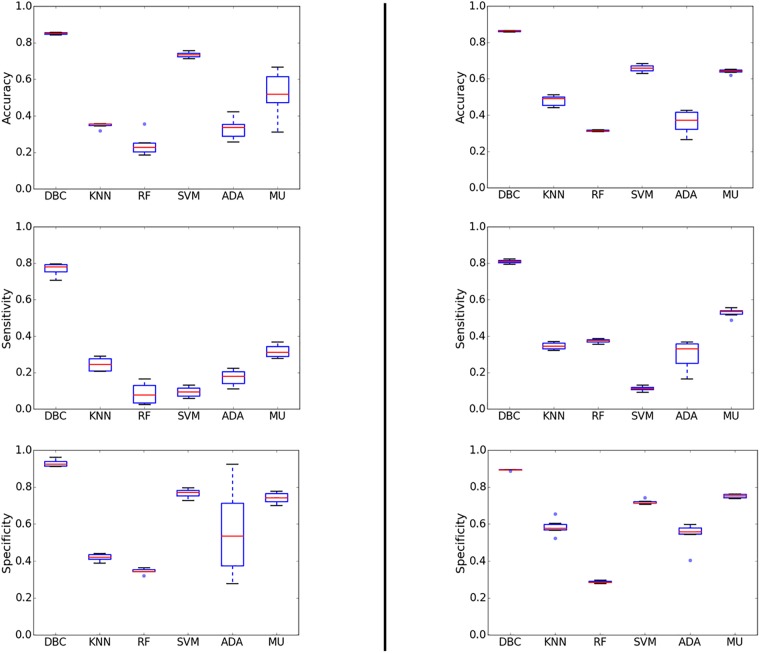
Accuracy, sensitivity and specificity of our algorithm (DBC), K-Nearest Neighbors (KNN), Random Forest (RF), Support Vector Machine (SVM), AdaBoost (ADA) and Mean based method (MU) at prediction window of 120 minutes, averaged over 144 choices of [O,T] settings and over (LEFT:) 3683 patients (online)/ (RIGHT:) 5–fold cross validation (offline).

We show the boxplots for the sensitivity, specificity and accuracy obtained at a prediction window of 120 minutes (2 hours) in both offline and online modes, averaged over 144 [O,T] settings in Fig 4. Note that in these 144 experiments, even the lowest accuracy achieved by our algorithm is higher than the highest accuracy of any other algorithm. Results for other values of prediction window are similar.

#### Algorithm analysis

Algorithm DBC is a hybrid approach that combines margin-based and distance-based classification methods. As described in section 4, by learning two boundaries DBC finds an uncertainty region between the boundaries and uses two different classification rules depending on whether a test case falls within or outside the uncertainty region. We now evaluate the benefit of this approach by comparing the performance of DBC with a margin-based classifier without the uncertainty region and a purely distance-based approach that does not use the dual boundaries of DBC—that is same as KNN. Table 2 shows the results over all the 864 [O,P,T] window settings: averaged for 3683 patients in the online mode and over 5-fold cross validation in the offline mode as described above.

**Table 2:** Mean (standard deviation in parentheses) of performance of algorithms—Our (DBC), KNN, Linear SVM—Averaged over all the 864 [O,P,T] window settings: Averaged for 3,683 patients in the online mode and over 5-fold cross validation in the offline mode.

	Algorithm	DBC	KNN	Linear SVM
Online	Sensitivity	**0.77 (0.04)**	0.31 (0.05)	0.11 (0.05)
	Specificity	**0.92 (0.02)**	0.47 (0.03)	0.83 (0.05)
	Accuracy	**0.85 (0.01)**	0.40 (0.03)	0.73 (0.05)
Offline	Sensitivity	**0.83 (0.01)**	0.34 (0.02)	0.17 (0.03)
	Specificity	**0.90 (0.01)**	0.56 (0.04)	0.85 (0.02)
	Accuracy	**0.87 (0.01)**	0.46 (0.03)	0.78 (0.04)

The goal of the hybrid approach of DBC is to learn the two boundaries such that we have very good classification performance outside the uncertainty region. This hypothesis is confirmed in our experiments. Table 3 shows the classification performance of DBC (for the offline case, as shown in Tables 2 and 3 above) in each of the three regions of the feature space (see Fig 3): AHE, Non-AHE and Uncertainty Region. We observe that the performance of DBC is indeed much higher outside the uncertainty region.

**Table 3:** Mean performance of algorithm DBC in the three regions learned—Averaged over all the 864 [O,P,T] window settings: Over 5-fold cross validation in the offline mode.

	AHE	Non-AHE	Uncertainty
Sensitivity	0.93	0.96	0.73
Specificity	0.97	0.99	0.82
Accuracy	0.96	0.98	0.79

### 5.6 Addition of vitals

We study the performance of all the classifiers when additional vitals—HR, PUL, RR, and OSAT—are used. Table 4 shows the performance averaged over 5-fold cross validation, over all 864 choices of [O,P,T] window settings. The uppermost rows show the results of using (1) only MAP measurements. The other rows (downwards) show the results of using (2) MAP and HR, (3) MAP, HR and PUL, (4) MAP, HR, PUL and RR and (5) MAP, HR, PUL, RR and OSAT respectively.

**Table 4:** Mean (standard deviation in parentheses) of performance of algorithms—Our (DBC), KNN, RF, SVM, ADA and MU—Averaged over all the 864 [O,P,T] window settings using 5-fold cross validation. The topmost rows show the results of using (1) only MAP measurements. The remaining rows show the results of using (2) MAP and HR, (3) MAP, HR and PUL, (4) MAP, HR, PUL and RR and (5) MAP, HR, PUL, RR and OSAT respectively. Best results in each row are indicated in bold.

Vitals	Algorithm	DBC	KNN	RF	SVM	ADA	MU
MAP	Sensitivity	**0.83 (0.01)**	0.34 (0.02)	0.43 (0.03)	0.19 (0.03)	0.39 (0.11)	0.59 (0.03)
	Specificity	**0.90 (0.01)**	0.56 (0.04)	0.34 (0.03)	0.78 (0.03)	0.57 (0.09)	0.79 (0.02)
	Accuracy	**0.87 (0.01)**	0.46 (0.03)	0.37 (0.03)	0.74 (0.04)	0.44 (0.08)	0.69 (0.02)
+ HR	Sensitivity	**0.78 (0.01)**	0.36 (0.05)	0.22 (0.04)	0.19 (0.03)	0.38 (0.10)	0.56 (0.1)
	Specificity	0.69 (0.01)	0.62 (0.05)	0.22 (0.04)	**0.82 (0.04)**	0.62 (0.11)	0.54 (0.1)
	Accuracy	**0.72 (0.01)**	0.51 (0.05)	0.22 (0.04)	0.61 (0.04)	0.44 (0.09)	0.55 (0.1)
+ PULSE	Sensitivity	**0.55 (0.005)**	0.45 (0.1)	0.22 (0.04)	0.25 (0.06)	0.45 (0.12)	0.29 (0.06)
	Specificity	0.54 (0.005)	0.44 (0.1)	0.21 (0.05)	**0.82 (0.06)**	0.61 (0.13)	0.45 (0.04)
	Accuracy	0.54 (0.003)	0.44 (0.1)	0.22 (0.04)	**0.66 (0.05)**	0.49 (0.10)	0.37 (0.04)
+ RR	Sensitivity	**0.61 (0.01)**	0.5 (0.05)	0.39 (0.05)	0.15 (0.05)	0.20 (0.05)	0.29 (0.06)
	Specificity	0.54 (0.01)	0.44 (0.05)	0.55 (0.02)	**0.84 (0.06)**	0.63 (0.12)	0.65 (0.05)
	Accuracy	**0.57 (0.005)**	0.47 (0.05)	0.47 (0.02)	0.51 (0.1)	0.33 (0.08)	0.52 (0.1)
+ OSAT	Sensitivity	0.46 (0.02)	**0.53 (0.03)**	0.37 (0.05)	0.14 (0.04)	0.21 (0.04)	0.26 (0.04)
	Specificity	0.72 (0.02)	0.44 (0.02)	0.53 (0.04)	**0.88 (0.04)**	0.67 (0.17)	0.68 (0.04)
	Accuracy	0.61 (0.01)	0.49 (0.02)	0.45 (0.03)	**0.7 (0.19)**	0.42 (0.11)	0.45 (0.06)

As additional vitals are used, the accuracy of our classifier reduces and the best results are obtained when only MAP measurements are used. With more vitals, SVM achieves comparable or higher than that of DBC. However, in the case of even SVM, the highest accuracy is obtained with the use of MAP measurements only. Overall, among all the different combinations of vitals tested, the best results are obtained by our algorithm when only MAP measurements are used. These results suggest that MAP measurements provide the most discriminatory feature to identify patients who are at risk of AHE.

### 5.7 Discussion

#### Why does our classification algorithm work well?

The answer lies in the data. We first observe that mean MAP measurements over an observation window is a good predictor of AHE. This has been validated by previous work as well [22]. Our experiments also show that addition of mean values of other vitals in our framework does not improve the predictive accuracy.

**Fig 5:**
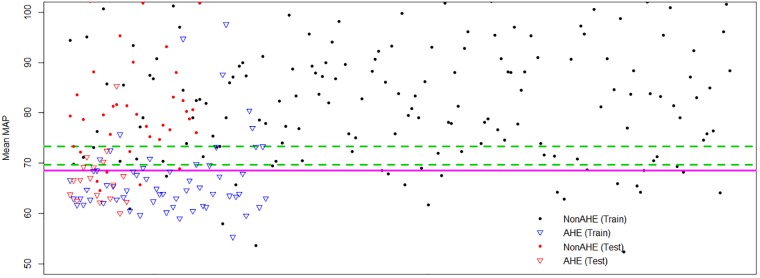
Mean MAP measurements (along Y-axis) of AHE and non-AHE patients in train and test data. Each point belongs to a single patient stretched out along the X-axis. Circles: Non-AHE, Triangles: AHE, Blue: Train data, Red: Test data, Red solid line: Classification boundary learned by SVM, Greed dashed lines: Dual classification boundaries learned by our classifier.

However, not all AHE patients show very low mean in their observation windows. Fig 5 shows the mean MAP measurements of the patients in our dataset with different markers indicating division into train and test sets, as well as AHE and non-AHE data. The boundary obtained by a linear SVM classifier and the dual boundaries obtained by our classifier on the training data are also shown.

While it is true that most of the AHE datapoints (triangle markers) lie below the SVM boundary, with low mean MAP, there are several above the boundary as well. Our algorithm gives special consideration to some of the datapoints that are between our two boundaries—the region we call the uncertainty region. These datapoints are difficult to classify into AHE or non–AHE by using a single boundary. Hence we use a distance based strategy to classify these datapoints.

The data in the uncertainty region mostly belong to patients whose MAP measurements were observed to fluctuate considerably in the observation window. For example, in some cases, the BP remains in the normal range initially and becomes low and again comes back to the normal range after some time. In such cases, the mean is often close to the decision boundary and a single boundary classifier tends to misclassify them. These were the cases that were better classified by finding the distance from the mean of the training data in each class.

#### Design choices

Our hybrid approach utilizes a combination of margin-based and distance-based classification methods. A detailed analysis of the differences between our approach and these approaches is presented in section 4. Previous work in AHE prediction (described in section 2.2) have used these and other standard classifiers. Our work has focussed more on algorithmic development and lesser on feature design. Our choice of mean–based feature was primarily motivated by its performance demonstrated in previous studies [22]. An advantage of using only mean and standard deviation of all measurements, is that there is no restriction on the number of measurements or duration in which these measurements are taken for the observation or test windows in our classifier. This is useful since the number of measurements and length of stay varies considerably across patients. This also avoids the problems of missing values and irregular sampling of vitals within and across patient records that often needs to be addressed if other features are used. There is a growing body of literature on feature design from clinical time series (e.g. [37]) and further studies on the effectiveness of other features would be useful.

#### Limitations and future work

Features from other clinical data have been used for AHE prediction (e.g. [30]) but a large-scale evaluation has not been done. In our study, we evaluate the performance of classification on addition of mean values of other vitals as features. We find no improvement over the performance achieved when only mean MAP values are used. This conclusion, although not surprising since we are predicting a condition of sustained low blood pressure, is limited to the feature design and classification framework used in our experiments. For example, we train our classifier for each vital separately and use a majority voting scheme to determine the final label, but many other approaches are possible using the DBC classifier itself to combine data from different vitals. Performance of DBC on features that combine measurements from vitals have also not been tested.

The DBC classifier was designed specifically for AHE prediction. However it could be applicable in other contexts with similar data characteristics with respect to the vital measurements. This remains to be explored.

Acute Hypotensive Episodes can be caused by a variety of clinical conditions. This study, similar to many previous works on predictive models for AHE prediction, remains oblivious to the underlying causes and patient diversity. It would be useful to study predictive models for AHE for clinically meaningful groups based on patient characteristics, potentially leading to hierarchical models. MIMIC II data is limited to a single tertiary hospital and multi-center studies are needed to further validate the findings of this study.

## 6 Conclusion

We present a new method to identify patients at risk of Acute Hypotensive Episodes (AHE) in critical care. Our method uses a first-of-its-kind approach of using a dual–boundary classifier whose design is motivated by characteristics of the Mean Arterial Pressure (MAP) measurements observed in critical care patients. We perform extensive experiments using data of more than 4,500 patients from the MIMIC II database in more than 850 different experimental settings. Our algorithm, that can be used in both online and offline manner, is compared with the best known classifiers for AHE prediction and is shown to significantly outperform them in predictive accuracy. In particular, we achieve nearly 80% sensitivity and 95% specificity while predicting 2 hours in advance of the onset of AHE.
